# *Mycobacterium tuberculosis* Complex Lipid Virulence Factors Preserved in the 17,000-Year-Old Skeleton of an Extinct Bison, *Bison antiquus*

**DOI:** 10.1371/journal.pone.0041923

**Published:** 2012-07-30

**Authors:** Oona Y-C. Lee, Houdini H. T. Wu, Helen D. Donoghue, Mark Spigelman, Charles L. Greenblatt, Ian D. Bull, Bruce M. Rothschild, Larry D. Martin, David E. Minnikin, Gurdyal S. Besra

**Affiliations:** 1 School of Biosciences, University of Birmingham, Edgbaston, Birmingham, United Kingdom; 2 Centre for Clinical Microbiology (M9), Royal Free Campus, University College London, London, United Kingdom; 3 Centre for the History of Medicine, University College London, London, United Kingdom; 4 Kuvin Center for the Study of Infectious and Tropical Diseases and Ancient DNA, Hadassah Medical School, Hebrew University, Jerusalem, Israel; 5 Organic Geochemistry Unit, School of Chemistry, University of Bristol, Bristol, United Kingdom; 6 Department of Medicine, Northeast Ohio Medical University, Rootstown, Ohio, United States of America; 7 Biodiversity Institute, University of Kansas, Lawrence, Kansas, United States of America; Johns Hopkins University School of Medicine, United States of America

## Abstract

Tracing the evolution of ancient diseases depends on the availability and accessibility of suitable biomarkers in archaeological specimens. DNA is potentially information-rich but it depends on a favourable environment for preservation. In the case of the major mycobacterial pathogens, *Mycobacterium tuberculosis* and *Mycobacterium leprae*, robust lipid biomarkers are established as alternatives or complements to DNA analyses. A DNA report, a decade ago, suggested that a 17,000-year-old skeleton of extinct *Bison antiquus*, from Natural Trap Cave, Wyoming, was the oldest known case of tuberculosis. In the current study, key mycobacterial lipid virulence factor biomarkers were detected in the same two samples from this bison. Fluorescence high-performance liquid chromatography (HPLC) indicated the presence of mycolic acids of the mycobacterial type, but they were degraded and could not be precisely correlated with tuberculosis. However, pristine profiles of C_29_, C_30_ and C_32_ mycocerosates and C_27_ mycolipenates, typical of the *Mycobacterium tuberculosis* complex, were recorded by negative ion chemical ionization gas chromatography mass spectrometry of pentafluorobenzyl ester derivatives. These findings were supported by the detection of C_34_ and C_36_ phthiocerols, which are usually esterified to the mycocerosates. The existence of Pleistocene tuberculosis in the Americas is confirmed and there are many even older animal bones with well-characterised tuberculous lesions similar to those on the analysed sample. In the absence of any evidence of tuberculosis in human skeletons older than 9,000 years BP, the hypothesis that this disease evolved as a zoonosis, before transfer to humans, is given detailed consideration and discussion.

## Introduction

Tuberculosis was present from an early date in North America, but clear evidence for its distribution and origins is by no means complete [1], [2]. An intriguing aspect of ancient North American tuberculosis is its apparent prevalence in ice-age bovids and mastodons who may have nurtured the disease without being rapidly killed [3], [4]. Bone pathology of skeletons, collected in Natural Trap Cave (Wyoming), indicates the presence of such an ancient animal tuberculosis reservoir [5], [6]. Lesions suggestive of tuberculosis were seen in skeletons of bighorn sheep, musk ox and *Bison antiquus* [6]. In this bison, dated to 17,870±230 BP, it was possible to demonstrate ancient DNA characteristic of the *Mycobacterium tuberculosis* complex, confirming the oldest proven case of tuberculosis [7].

**Figure 1:**
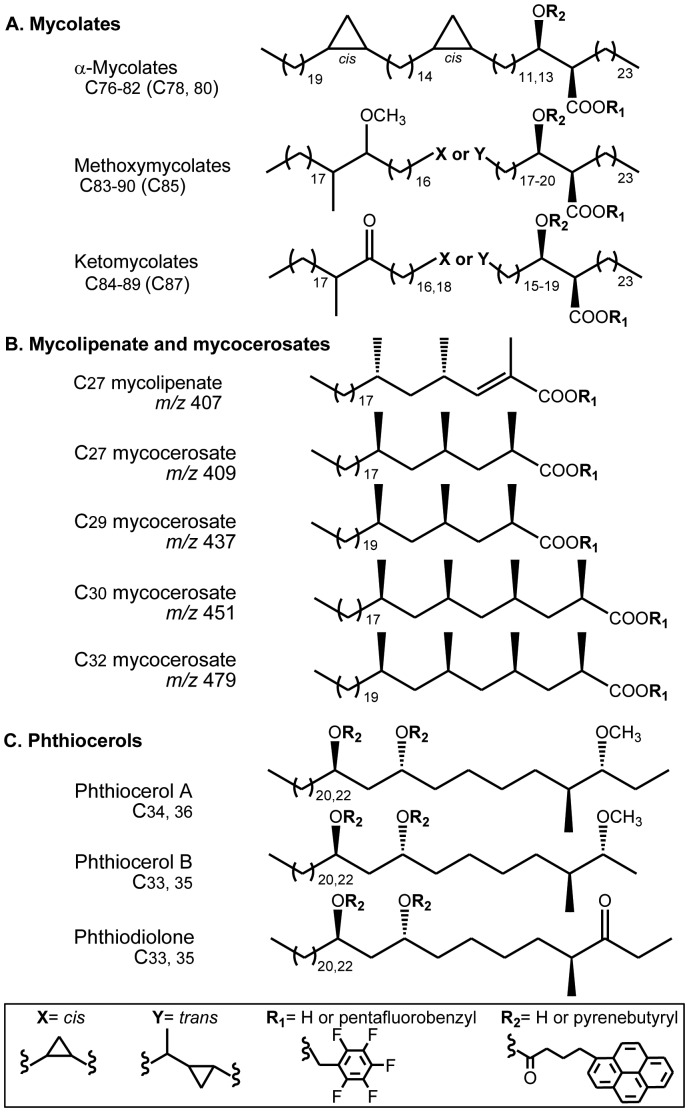
Lipid biomarkers for *M. tuberculosis*. **A.** Generalized structures of the α-, methoxy- and ketomycolates; the main components are in brackets. **B.** Structures of mycolipenate and mycocerosates, showing ions used for selected ion monitoring on NI-CI GC-MS analysis. **C.** Structures of members of the phthiocerol family.

The use of DNA can be complemented by other biomarkers, as reviewed recently [8], [9]. Mycolic acids (MAs) (Figure 1A) and phthiocerol dimycocerosate (PDIM) waxes (Figure 1B, C) are characteristic major components of the cell envelopes of *M. tuberculosis* [10], [11]. Using a combination of DNA and MA analysis, the oldest case of human tuberculosis infection was established in skeletons of a woman and adjacent child from Atlit-Yam (Israel) [12]. Analysis of the mycocerosate components (Figure 1B) of PDIM waxes was established in the investigation of a skeletal collection from Coimbra (Portugal) [13]. In the same study [13], another lipid biomarker, C_27_ mycolipenic acid (Figure 1B), was encountered in a minority of samples.

The present study investigated the same bone samples used previously [7], and recorded a weak mycolic acid profile. However, a clear profile of mycocerosic acids was obtained, supported by the identification of phthiocerol components. In addition, a strong presence of mycolipenic acids was recorded. These findings independently document the presence of tuberculosis in this ancient extinct bison skeleton and demonstrate the long-term stability of these lipid virulence factors. The confirmation of tuberculosis in an animal bone much older than any positive human skeletons raises the possibility that the ancient evolution of the disease was as a zoonosis.

## Results and Discussion

### Bone Samples and Extraction of Lipids

The two bone samples analysed were from an extinct bison (*Bison antiquus*) buried in sediments, dated to 17,870±230 years BP, in Natural Trap Cave (Wyoming) [7]. The first sample (“Bison 1”) originated from the undermined articular surface of a metacarpal but the second sample (“Bison 2”) was from a site on the same bone remote from this lesion [7]. The material was exactly the same as that used for the previous ancient tuberculosis DNA analyses [7].

Bone samples were hydrolysed by a protocol designed to release all the long-chain lipid components [9], [12], [13] and the acidic components were converted to pentafluorobenzyl (PFB) esters [12], [13]. The extract was fractionated into three distinct lipid classes, containing non-hydroxylated fatty acid PFB esters, mycolic acid PFB esters and underivatised phthiocerols [12], [13]. The latter two classes were allowed to react with pyrenebutyric acid (PBA) to produce PBA-PFB mycolates and di-PBA derivatives of the phthiocerol family (Figure 1). These fluorescent derivatives were analysed by sequential combinations of reverse and normal phase high performance liquid chromatography (HPLC) [12]. The non-hydroxylated fatty acid PFB esters were fractionated further to produce material enriched in PFB esters of mycocerosic and mycolipenic acids, which were analysed by negative ion chemical ionization gas chromatography mass spectrometry (NICI-GCMS) and selected ion monitoring (SIM) [13].

### Detection of Mycolic Acids

**Figure 2:**
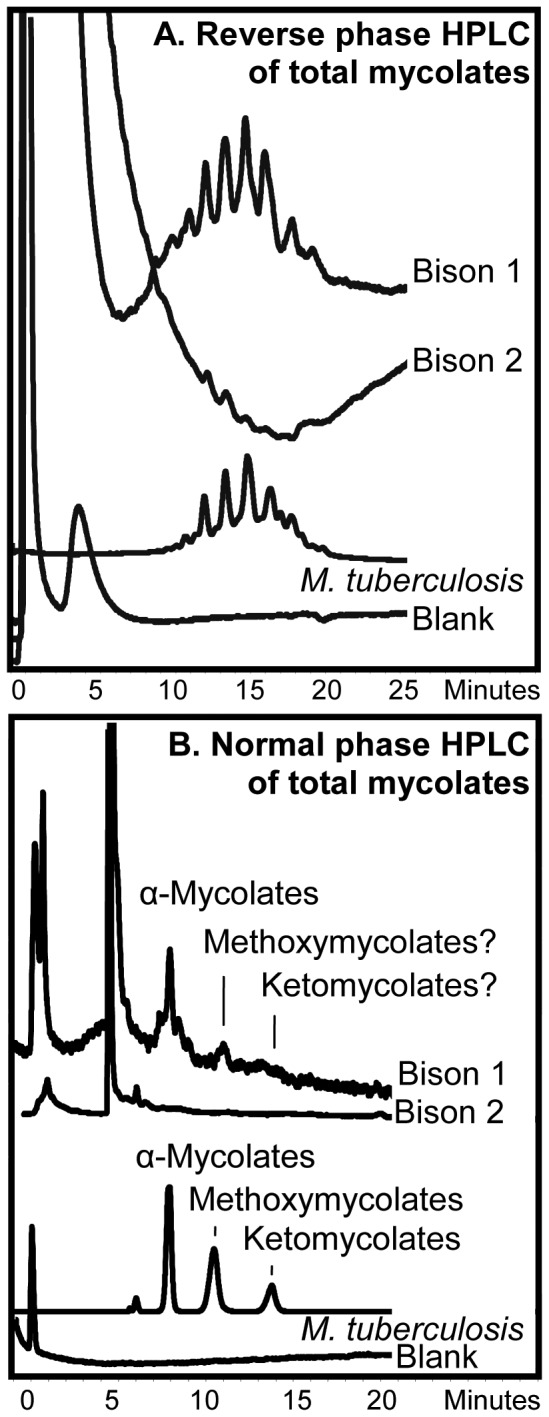
Fluorescence HPLC of pyrenebutyric acid derivatives of pentafluorobenzyl esters of total mycolic acids. The derivatives analysed were from Bison 1 and Bison 2 bones and standard *M. tuberculosis*. **A.** Reverse phase HPLC of total mycolates. **B.** Normal phase HPLC of total mycolates collected from reverse phase separations.

**Figure 3:**
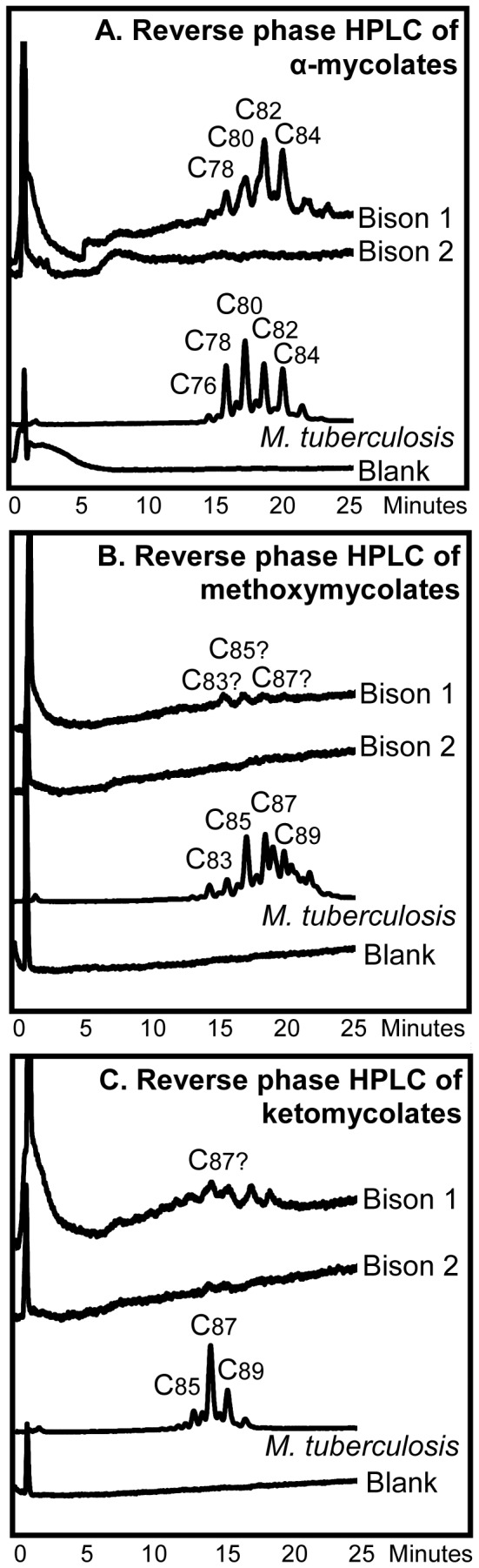
Reverse phase fluorescence HPLC of pyrenebutyric acid derivatives of pentafluorobenzyl esters of mycolic acid classes. The derivatives analysed were from Bison 1 and Bison 2 bones and standard *M. tuberculosis*. The analyzed fractions were collected from the normal phase separation (Figure 2B). **A.** α-Mycolates. **B.** Methoxymycolates. **C.** Ketomycolates.

Reverse phase HPLC of the PBA-PFB mycolate fractions indicated the presence of long-chain mycolates in sample Bison 1, but the profile for Bison 2 was very weak (Figure 2A). The total components in the region corresponding to mycolates, from the reverse phase HPLC of Bison 1 and 2 extracts, were collected and analysed by normal phase HPLC (Figure 2B). A recognizable profile was recorded for Bison 1, with the α-mycolates being the most abundant. However, the signal was not clean, with minor additional peaks eluting before and after the main component (Figure 2B). Methoxymycolates were apparently present in low amounts, but there was only a weak indication for ketomycolates. In the normal phase HPLC profile of the total mycolates from Bison 2 no clear peaks could be discerned. The fractions, corresponding to the positions of α-mycolates, methoxymycolates and ketomycolates (Figure 2B), were collected and re-analyzed by reverse phase HPLC (Figure 3). An informative profile was obtained for the α-mycolates from Bison 1 but nothing was observed for Bison 2 (Figure 3A). Weak indications of methoxymycolates and ketomycolates were only discernible in Bison 1 (Figure 3B, C).

### Detection of Mycocerosic and Mycolipenic Acids

**Figure 4:**
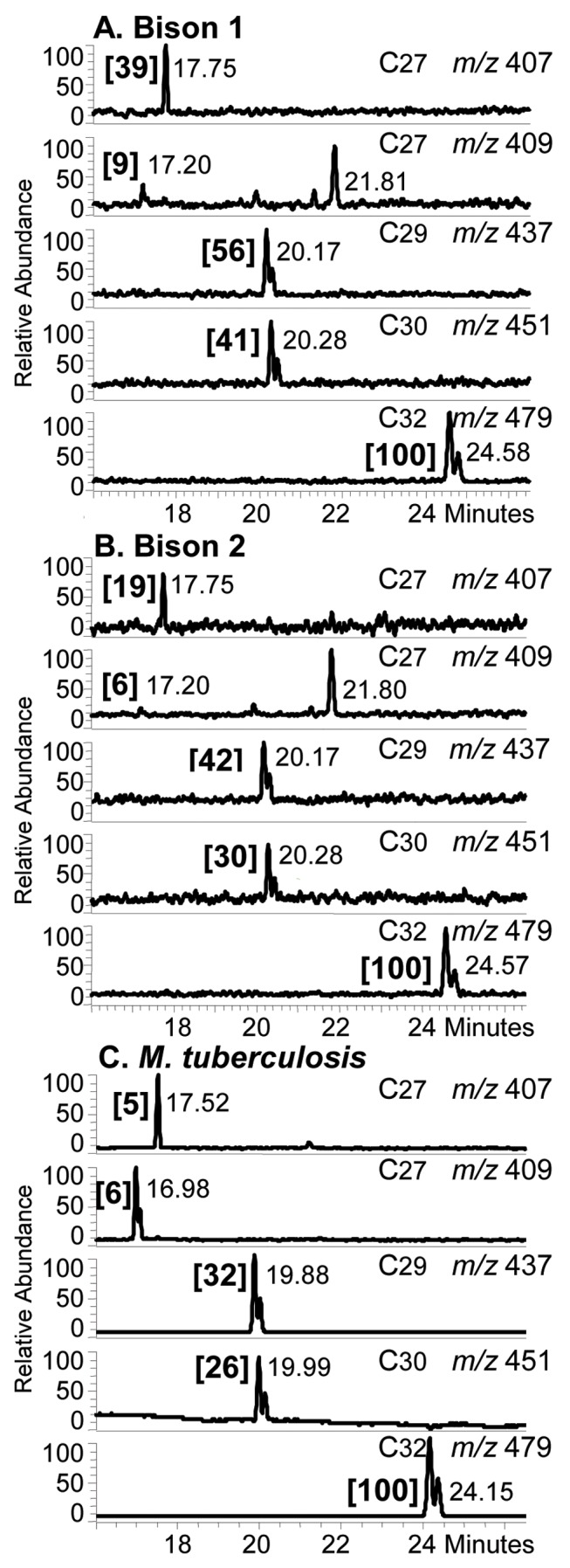
Selected ion monitoring NI-CI GC-MS of mycolipenic and mycocerosic acid pentafluorobenzyl fractions. **A.** Bison 1. **B.** Bison 2. **C.** Standard *M. tuberculosis.* The *m/z* 407 (mycolipenate), 409, 437, 451 and 479 (mycocerosates) ions correspond to the components shown in Figure 1B. The intensities of the mycocerosate and mycolipenate peaks, in square brackets, are normalized to that [100] of the major C_32_ mycocerosate. It was not possible to record all three profiles on the same occasion, so the retention times for the standard *M. tuberculosis* extract do not correspond exactly. In profiles **A** and **B**, the C_27_ *m/z* 409 peaks at 21.81 and 21.80, respectively, correspond to straight-chain heptacosanoate.

Positive profiles of C_29_, C_30_ and C_32_ mycocerosates were recorded by SIM NICI-GCMS for both Bison 1 and 2 (Figure 4). Additionally, lesser proportions of C_27_ mycocerosates were distinguishable, but no significant proportions of C_33_ and C_34_ mycocerosates were encountered (**Figure S1**). The mycocerosates were observed as characteristic double peaks, due to partial racemization during the alkaline hydrolysis [13]. Positive recognition of the *M. tuberculosis* complex mycocerosate pattern was confirmed by the GC retention time values, particularly the overlapping of the C_29_ and C_30_ components (Figure 4). This highly diagnostic chromatographic behaviour is a result of the tetramethyl-branched C_30_ mycocerosate being relatively more volatile in comparison with the trimethyl-branched C_29_ ester [9], [13]. In both Bison 1 and 2, a substantial proportion of C_27_ mycolipenate was observed (Figure 4) as a single peak, racemization not being possible [9], [13]. The identification of C_27_ mycolipenate was also confirmed by comparison of the GC retention time with an authentic standard (Figure 4) and correlation with a previous detailed study [13]. The full range of positive and negative NICI-GCMS mycocerosate and mycolipenate profiles is shown in **Figure S1.**

### Detection of Phthiocerol Family

**Figure 5:**
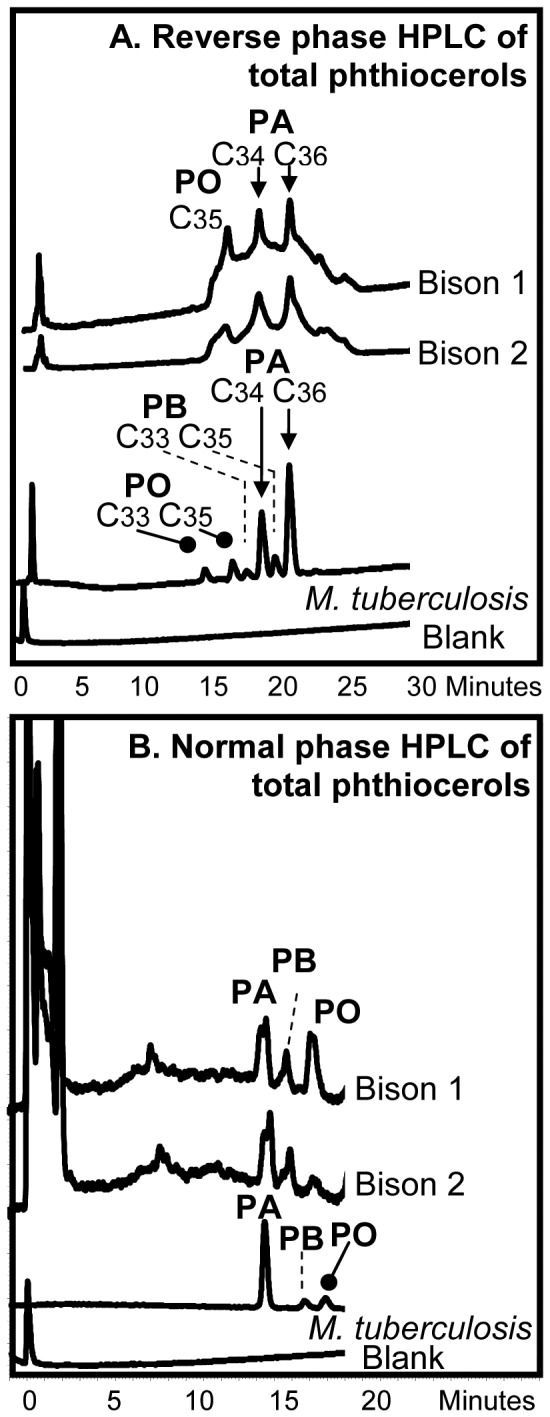
Fluorescence HPLC of di-pyrenebutyric acid derivatives of members of the phthiocerol family. The derivatives analysed were from Bison 1 and Bison 2 bones and standard *M. tuberculosis*. **A.** Reverse phase HPLC of total phthiocerol family fraction. **B.** Normal phase HPLC of phthiocerol family derivatives collected from reverse phase separations. Abbreviations: **PA**, phthiocerol A; **PB**, phthiocerol B; **PO**, phthiodiolone.

Reverse phase HPLC of the PBA phthiocerol fraction from Bison 1 and 2 both showed components corresponding to C_34_ and C_36_ phthiocerol A and C_35_ phthiodiolone (Figure 5A). The collected total PBA phthiocerol family fractions were analyzed by normal phase HPLC (Figure 5B), providing profiles with components corresponding to phthiocerols A and B and phthiodiolone. The separated fractions from the normal phase analysis (Figure 5B) were subjected to reverse phase HPLC, but there was insufficient material for profiles to be registered.

### Significance of the Presence of Lipid Biomarkers

The presence of mycolic acids, having an overall size similar to those found in *M. tuberculosis* (Figure 2A), is highly significant, providing confirmation of the previous recovery of DNA characteristic of the *M. tuberculosis* complex [7]. In that study [7], spoligotyping indicated that the infection was not due to *M. bovis*, but *M. tuberculosis* or *M. africanum* were possibilities. Not surprisingly, the bone sample taken from near the observed lesion (Bison 1) gave the best profile, but there was a very faint response for Bison 2 (Figure 2A). The normal phase HPLC examination of the collected mycolates (Figure 2B) gave confirmatory evidence for α-mycolates in Bison 1, with one good sharp peak accompanied by some possibly degraded components (Figure 2B). The Bison 1 extract also had a broad peak, possibly representing methoxymycolates, and a slight elevation of the baseline might be interpreted as a suggestion of ketomycolates (Figure 2B). If diagenetic modification of mycolates takes place, it is probable that the more hydrophilic and chemically reactive ketomycolates are more susceptible, followed by the intermediate methoxymycolates and the relatively hydrophobic α-mycolates. For Bison 2, the normal phase HPLC analysis of total mycolates was negative (Figure 2B). On subjecting total α-mycolates, collected from the normal phase HPLC analyses (Figure 2B), to reverse phase HPLC an acceptable profile was recorded for Bison 1 (Figure 3A). This profile (Figure 3A) corresponds to the standard, but the peaks are broadened by possible degradation. It is also encouraging that a very weak series of peaks can be discerned for methoxymycolates and ketomycolates in Bison 1 (Figure 3B, C). The above evidence points to the presence of mycobacterial mycolic acids, but a clear diagnosis of *M. tuberculosis* complex infection cannot be made solely on these data.

Of greater significance is the discovery of pristine C_29_, C_30_ and C_32_ mycocerosates and C_27_ mycolipenate (Figure 4) and the C_34_ and C_36_ phthiocerols A (Figure 5). This combination of mycocerosates is typical of *M. tuberculosis sensu stricto* [11], [13], as is the presence of comparable amounts of the C_34_ and C_36_ phthiocerols. A limited study [14] indicated that *M. bovis* usually has an enhanced proportion of C_27_ mycocerosate and a preponderance of C_34_ over C_36_ phthiocerols, when compared with *M. tuberculosis sensu stricto*. It is interesting that the proportion of phthiodiolone in Bison 1 is comparable to that of phthiocerol A. For Bison 2, however, phthiodiolone is reduced in proportion; this indicates that this “keto” component may have experienced some selective degradation as for ketomycolates (Figure 2B). The normal phase HPLC peaks (Figure 5B) for phthiocerol A appeared to be doublets, suggesting some diagenetic racemization. The presence of both mycocerosates and phthiocerols in the same extract suggests that intact PDIM waxes were present in these ancient bones. These PDIM waxes are particularly robust, surviving acid methanolysis and aqueous alkaline hydrolysis [14], [15]. It is not surprising, therefore, that they are apparently recovered intact from such an ancient source, when mycolic acids are diminished in abundance (Figures 2, 3, 4, 5). If PDIMs can survive intact for ∼17,000 years, it appears possible that, in favourable circumstances, they might be found in archaeological material way back into antiquity.

It is more surprising, however, that clear evidence for mycolipenic acid was observed in both samples. Mycolipenic acid [11], [13] is a constituent of characteristic glycolipids [16], the penta-acyl trehaloses (PATs) [16], [17]. Mycolipenates appear to be limited in distribution to virulent strains of members of the *M. tuberculosis* complex, such as *M. tuberculosis, M. bovis* and *M. africanum* [17]. It might be expected that the trehalose unit would render PATs susceptible to degradation compared to the non-polar PDIM waxes. However, the PATs are also relatively non-polar [16] and hydrophobic, due to the presence of long-chain fatty acid substituents on five out of the eight trehalose sites [17]. Mycolipenates may survive in this protected hydrophobic environment, although there is currently no evidence for the survival of intact PAT glycolipids. Another possibility is that the conjugated αβ-unsaturated unit in the mycolipenate [Figure 1B] confers additional resistance to diagenesis.

*M. tuberculosis* produces a class of relatively polar diacylated trehalose (DAT) glycolipids, with mycolipanolic and mycosanoic (2,4-dimethyldocosanoic) acid components [11], [16], [18]. Mycolipanolate was not investigated, but no evidence was obtained by NICI-GCMS for an ion at *m/z* 367, corresponding to C_24_ mycosanoate (**Figure S1)**. It appears, therefore, that mycolipenic acid (Figure 1B) is an informative biomarker for ancient tuberculosis. Mycolipenate was found in low abundance in only 9 out of 37 mycocerosate-positive skeletons from the more-recent Coimbra collection [13]. Mycolipenates also have value in distinguishing ancient tuberculosis and leprosy, as they are not synthesized by *Mycobacterium leprae* [11].

The previous aDNA results [7] indicated that the infection did not correlate with bovine tuberculosis, but could be due to *M. tuberculosis* or *M. africanum.* The present lipid results do not contradict this conclusion. In particular, the mycocerosate profiles are indicative of *M. tuberculosis*, as only small proportions of C_27_-mycocerosate were recorded (Figure 4). In previous studies, it was shown that extracts of *M. bovis* had relatively enhanced amounts of this component [14]. The profiles for members of the phthiocerol family (Figure 4) also resemble those for *M. tuberculosis* more closely than those for *M. bovis,* with a predominance of C_36_ phthiocerol A over the C_34_ component [14]. The degraded mycolate profiles (Figs. 2 and 3) are not particularly informative, but the size of the α-mycolates is compatible with those from members of the *M. tuberculosis* complex. The presence of C_27_-mycolipenate is not diagnostic, as this component is found in various virulent members of the *M. tuberculosis* complex [17]. The overall conclusion is that the infection was by a member of the *M. tuberculosis* complex, with *M. bovis* being an unlikely candidate.

Looking beyond the obvious paleopathological significance of the present results, it should be noted that, to our knowledge, these MAs and PDIMs are the oldest recognizable virulence factors ever recorded for tuberculosis, or perhaps any other infectious disease. The importance in virulence of these two lipid classes can be attributed to at least two features of these compounds. Firstly, both the MAs and PDIMs are considered to be integral components of the cell envelope architecture of *M. tuberculosis* [10], [11]. In *M. tuberculosis,* the intense hydrophobicity of the outer membrane may contribute to the defences of the pathogen in resisting attack by infected host cells. A wide range of biological activities have been associated with the lipids identified in this study and these have been comprehensively reviewed [19]–[21]. In particular, there is evidence that both MAs and PDIMs are actively exported with a direct influence in generating foamy macrophages and granulomas, contributing to the disease process [22]–[25]. These exported lipids may accumulate in bone matrices, thereby enhancing their preservation and eventual detection. Although the virulence factor activity of these lipids has been demonstrated for modern human tuberculosis, it is likely that the lipids would have a similar role in ancient animal disease.

### Implications for the Evolution of Tuberculosis

Great strides are being made in the paleogenomics of tuberculosis [26]–[36], particularly in unravelling the complexities of the interrelationships between relatively modern variants responsible for current disease [30], [31]. However, the early evolutionary pathways, defining exactly from where and how tuberculosis originated, remain indistinct. The challenge is to chart a pathway from ancestral environmental freely-circulating mycobacterial species to *M. tuberculosis sensu stricto*, which is an obligate pathogen with no environmental niche. Currently favoured hypotheses all point to an evolutionary bottle-neck, estimated to have been around 35,000 BP [27], [28], [35], [37]. Subsequent to this time period, the evolution of a range of particular clades follows an almost linear clonar evolutionary pattern, with key deletions leading to the well-defined modern *M. tuberculosis* complex causing tuberculosis in humans and various animals [26]–[36], [38].

There is increasing evidence that, before reaching the discontinuity of the bottle-neck, extensive horizontal gene transfer (HGT) was taking place in ancestral tuberculosis strains [27], [38]–[41]. These pre-bottle-neck ancestral strains, sometimes termed *“M. prototuberculosis”* [28], [42], have been associated with the “smooth” colony-forming Canetti variants of *M. tuberculosis* [28], but “*Mycobacterium canettii*” isolates are not necessarily considered to be living representatives of the progenitor of the *M. tuberculosis* complex [32], [42], [43]. “*M. canettii*” strains can be regarded as a heterogeneous “out-group” whose evolution is distinct from other members of the *M. tuberculosis* complex [30], [31], [33]. “*M. canettii*” smooth strains continue to be encountered in isolated cases of tuberculosis, but they are usually confined to certain locations in the Horn of Africa [43], [44]. Inter human transfer is not known, but children and expatriates are more susceptible to infection, suggesting that the indigenous population has acquired significant immunity [44]. The geographical restriction, genetic diversity and specialised nutritional requirements of *“M. canettii”* isolates strongly favour an environmental reservoir [44]. It is not known if these bacteria can also be part of an animal reservoir in the Horn of Africa.

The positive identification of tuberculosis in this ancient bison skeleton establishes a clear beacon point in the historical record around which to explore an evolutionary scenario for tuberculosis in North America and elsewhere. Clearly recognisable human tuberculosis has not been recorded before 9,000 BP in Eurasia/North Africa [12], [34] and 2,100–1,900 BP in the Americas [1], [2], [45]. In an interesting, but isolated, report, tuberculosis was suggested to be a cause of possible endocranial paleopathology in a fossilized *Homo erectus* skeleton, dated 500,000 BP [46]; however, alternative interpretations have been suggested [47]. Apart from this single unconfirmed case [46], any clear indications for the presence of tuberculosis in very ancient human remains have not been reported. However, in the animal kingdom there are indications of widespread tuberculosis. In addition to the bison metacarpal, analysed in this study, 19% of 1,002 bovid specimens [3] and 52% of 113 mastodon bones [4] had similar lesions indicative of tuberculosis. The age range for the bovids is 125,000 to 8,000 BP [3] and the mastodon skeletons cover a range from 38,000 to 10,000 BP [4]. Bone lesions cannot be considered as complete proof of tuberculosis diagnosis, but the dearth of human bones with comparable lesions over the same time period of at least 100,000 years is very striking. This could be a consequence of the hunter-gatherer human population being thinly spread, whereas it may be easier to locate bones from large animal herds. A solution of this conundrum could simply be that *M. tuberculosis* was principally an animal disease during its early evolution, with transmission to humans occurring later. It has been noted previously [34], [35], [42] that such a scenario should not be dismissed.

### Conclusions

The highly sensitive analytical protocols employed have detected key tuberculosis lipid biomarkers in two samples from an extinct bison, lending solid support to previous aDNA conclusions [7]. Mycolic acids (Figure 1A) were indicated (Figs. 2 and 3), but the profiles demonstrated degradation and a positive diagnosis of tuberculosis could not be given. This contrasts with the very strong profiles (Figure 4) recorded for the mycocerosic acid components (Figure 1B) of the phthiocerol dimycocerosate (PDIM) waxes and, remarkably, for the mycolipenic acid (Figure 1B) component of pentaacyl trehaloses (PATs). Profiles (Figure 5) for components of the phthiocerol family (Figure 1C) suggested that intact PDIMs may have been present.

Conclusive evidence has been provided for the presence of tuberculosis in this ancient bison by detection of these lipid virulence factors. It has also been confirmed that tuberculosis may be identified in absence of macroscopically recognizable bone lesions and defects. Analysis of ancient DNA provides the most informative way to trace the evolution of tuberculosis, but the developing portfolio of diagnostic lipids, illustrated here, offers alternative routes to chart these evolutionary processes. The confirmation of tuberculosis in this exceptionally old 17,000 BP extinct bison and the current absence of any proven human tuberculosis older than 9,000 BP demands exploration of a hypothesis that tuberculosis may have originated and become established as a widespread zoonosis. Many, many more samples of potentially tuberculosis infected human and animal bones are urgently needed for analysis to support or disprove this or any other viable hypothesis.

## Materials and Methods

### Samples

The material, as used for the previous ancient tuberculosis DNA analyses [7], was from an extinct bison (*Bison antiquus*) buried in sediments, dated to 17,870±230 years BP, in Natural Trap Cave (Wyoming). The first sample (“Bison 1”; 13.5 mg) originated from the undermined articular surface of a metacarpal but the second sample (“Bison 2”; 13.0 mg) was from a site on the same bone remote from this lesion [7]. The metacarpal bone was stored in a dry sterile environment to avoid any possibility of external contamination. In the prevailing conditions of Natural Trap Cave, the possibility of external *post mortem* infection with *M. tuberculosis* is most unlikely. *M. tuberculosis* H37Rv was used to prepare standard profiles. Stringent precautions were taken against sample carry-over during the analyses. Essentially, this amounted to using new disposables for every analysis and running blanks between samples.

### Lipid Extraction

Samples were hydrolysed by heating with 30% potassium hydroxide in methanol (2 ml) and toluene (1 ml) at 100°C overnight [12], [13], [48], [49]. Long-chain compounds were extracted by a modification of a published method [13], substituting dichloromethane with toluene [48], [49] to ensure efficient extraction of the phthiocerols. The extract was treated with pentafluorobenzyl bromide, under phase-transfer conditions [12], [13], [48], [49], to convert acidic components into pentafluorobenzyl (PFB) esters. Subsequent fractionation on an Alltech 209250 (500 mg) normal phase silica gel cartridge [12], [13], [48], [49] gave fractions containing non-hydroxylated PFB esters, MA PFB esters and underivatized phthiocerols.

### Mycolic Acid Analysis

The MA PFB esters were reacted with pyrenebutyric acid (PBA) to produce PBA-PFB MA derivatives, which were purified on an Alltech 205250 (500 mg) C_18_ reverse phase cartridge [12], [48], [49]. The PBA-PFB mycolates were analysed by sequential reverse and normal phase HPLC, as described previously [12], [48], [49].

### Mycocerosic and Mycolipenic Acid Analysis

In a simplification of a previous protocol, which involved normal phase HPLC pre-purification [13], the non-hydroxylated PFB ester fraction was fractionated on an Alltech 205250 (500 mg) reverse phase silica gel cartridge, using a water-methanol/methanol/methanol-toluene elution sequence (**Figure S2**). A fraction enriched in mycocerosic acid and other longer chain (>C_20_) PFB esters was eluted by 100% methanol with the more usual C_12_ to C_20_ esters eluting in the earlier water/methanol fractions. The fraction containing possible mycocerosates and mycolipenates, was analysed by negative ion chemical ionization gas chromatography mass spectrometry (NICI-GCMS), as previously described [13] (**Figure S1**). PFB esters, on NICI-GCMS, fragment to produce negative carboxylate [M – H]^-^ ions, which can be detected at high sensitivity. Selected ion monitoring (SIM) was used to search for mycocerosate carboxylate ions at *m/z* 367.6311 (C_24_), 395.6844 (C_26_), 409.7111 (C_27_), 437.7645 (C_29_), 451.7911 (C_30_), 479.8445 (C_32_), 493.8712 (C_33_) and 507.8978 (C_34_). Additionally, *m/z* 407.6952 was monitored for the presence of the C_27_ mycolipenate carboxylate ion. Partial racemisation of mycocerosates during the alkaline hydrolysis leads to the formation of diasteroisomers, which resolve on gas chromatography to give characteristic doublets; in contrast, mycolipenates are singlets as they cannot racemise [13].

### Phthiocerol Family Analysis

In a new procedure, the phthiocerol fraction was converted to PBA esters by reaction with pyrenebutyric acid, under the same conditions used to derivatize MA PFB esters [12], [48], [49]. The crude phthiocerol di-PBA esters were purified by an Alltech 205250 (500 mg) C_18_ reverse phase cartridge, utilizing combinations of water, acetonitrile and dichloromethane; the PBA phthiocerols eluted in 100% acetonitrile and acetonitrile/dichloromethane 54∶6 and 48∶12 fractions (**Figure S3**). Reverse phase HPLC was performed on an Alltech 81412 Alltima C_18_ column (3 µ, 4.6 mm×50 mm) column in a VWR Hitachi Elite LaChrom HPLC linked to an L-2480 fluorescence detector, utilizing a gradient of acetonitrile/tetrahydrofuran, from 85∶15 to 60∶40 in 30 min (**Figure S4**). The fraction, corresponding to derivatives of members of the phthiocerol family, was collected and analyzed by HPLC on normal phase columns (Alltech 81414 Alltima Silica, 3 µm 50×4.6 mm). Eluting with heptane/ethyl acetate 99∶1 for 1 min, was followed by gradient of heptane/ethyl acetate 99∶1 to 91∶9 over 30 mins (**Figure S4**).

## Supporting Information

Figure S1 **Complete results of selected ion monitoring (SIM) negative ion-chemical ionisation gas chromatography-mass spectrometry (NICI-GCMS) analysis of pentafluorobenzyl (PFB) ester fractions, corresponding to mycocerosates and mycolipenates.**
(DOC)

Figure S2 **Solvent system for the purification of pentafluorobenzyl (PFB) mycocerosates on C_18_ reverse phase cartridges.**
(DOC)

Figure S3 **Solvent system for the purification of pyrenebutyric acid (PBA) derivatives of members of the phthiocerol family on C_18_ reverse phase cartridges.**
(DOC)

Figure S4 **HPLC conditions for analysis of pyrenebutyric acid (PBA) derivatives of members of the phthiocerol family.**
(DOC)
