# Supplementary material for: Mycobacterium tuberculosis Complex Lipid Virulence Factors Preserved in the 17,000-Year-Old Skeleton of an Extinct Bison, Bison antiquus
Source: PLoS One. 2012 Jul 30;7(7):e41923. doi: 10.1371/journal.pone.0041923 (PMC3408397; doi:10.1371/journal.pone.0041923)
Supplement: Figure S1 — Complete results of selected ion monitoring (SIM) negative ion-chemical ionisation gas chromatography-mass spectrometry (NICI-GCMS) analysis of pentafluorobenzyl (PFB) ester fractions, corresponding to mycocerosates and mycolipenates. (DOC) [file pone.0041923.s001.doc]

**Figure S1** Complete results of selected ion monitoring (SIM) negative ion-chemical ionisation gas chromatography-mass spectrometry (NICI-GCMS) analysis of pentafluorobenzyl (PFB) ester fractions, corresponding to mycocerosates and mycolipenates.

Instrumental conditions

The mass spectrometer (University of Bristol) was a [ThermoFinnigan MAT95 XP-Trap](http://www.bris.ac.uk/nerclsmsf/instruments/mat95.html), fitted with a Phenomenex Zebron ZB-5 (5% phenyl, 95% dimethylpolysiloxane) capillary column (30 m × 0.25 mm i.d. × 0.25 µm film thickness) using He as carrier gas (constant flow mode 1ml min^-1^) and ammonia as the CI reagent gas. A GC oven temperature gradient from 200 to 300 ⁰C at 6.7 ⁰C min^-1^ was used, the final temperature being held for 20 min. The ion source temperature was 250 ⁰C, the injector 300 ⁰C and the transfer line 300 ⁰C. Selected ion monitoring (SIM) was used for mycocerosate ions at *m/z* 367.6311 (C_24_), 395.6844 (C_26_), 409.7111 (C_27_), 437.7645 (C_29_), 451.7911 (C_30_), 479.8445 (C_32_), 493.8712 (C_33_) and 507.8978 (C_34_); additionally, *m/z* 407.6952 was monitored for the presence of C_27_ mycolipenic acid. The monoisotopic SIM *m/z* values were calculated using the LIST function of the ThermoFinnigan XCalibur software suite (version 1.3.2), which was also used to collect and analyse the data. The precision of the instrument was of the order of two decimal places, in the region of +/- a few hundred parts per million; however, the acquisition programme required SIM *m/z* values with four decimal places. The mass tolerance was set at a value of 0.15 amu. The dried samples were dissolved in 60 µl heptane and 1 µl was injected for analysis. Several heptane blanks were analysed between samples until a flat baseline was recorded.

Notes for recorded spectra

In each spectrum, the largest component has a Relative Abundance of 100.

The data shown above each peak are Retention Times (RT) and peak area (AA).

The mycocerosate peaks are characteristic doublets, with the first eluting component being the native mycocerosate followed by the racemised product. For the strong signals given by the C_29_, C_30_ and C_32_ mycocerosates, retention times and areas were recorded for both components of the doublets, but this was not possible for the weak signal of the C_27_ mycocerosate.

The C_27_ mycolipenate peak is a singlet, as racemization is not possible.

The most intense C_32_ mycocerosate peak at *m/z* 479 was given a value of 100% and the intensities of all other mycocerosate and mycolipenate peaks were normalized to that; the values are shown in brackets after the names Bison Sample 1 and Bison Sample 2.

**C_24_ *m/z* 367**

**Bison Sample 1 (0%)**

Time (min)

D:\Update\...\0235lsmsf_bison1

11/13/2009 11:06:22 AM

Bison 1

RT:

12.00 - 35.00

SM:

7B

12

14

16

18

20

22

24

26

28

30

32

34

0

10

20

30

40

50

60

70

80

90

100

Relative Abundance

17.02

16.58

17.74

30.71

27.59

34.90

20.43

22.19

24.45

31.35

28.48

13.31

18.74

14.93

26.64

NL:

2.56E6

m/z=

367.13-

368.13

MS

0235lsmsf_

bison1

**Bison Sample 2 (0%)**

Time (min)

D:\Update\...\0237lsmsf_bison2

11/13/2009 12:47:29 PM

Bison 2

RT:

12.00 - 35.00

SM:

7B

12

14

16

18

20

22

24

26

28

30

32

34

0

10

20

30

40

50

60

70

80

90

100

Relative Abundance

17.02

22.66

27.54

34.32

19.24

21.89

26.34

25.51

32.23

16.04

18.49

31.19

28.87

14.83

12.14

NL:

1.62E6

m/z=

367.13-

368.13

MS

0237lsmsf_

bison2

NOTES

Assignment of peaks, with retention time(s) (RT) (min)

RT: 17.02 Tetracosanoate

**C_26_ *m/z* 395**

**Bison Sample 1 (0%)**

D:\Update\...\0235lsmsf_bison1

11/13/2009 11:06:22 AM

Bison 1

RT:

12.00 - 35.00

SM:

7B

12

14

16

18

20

22

24

26

28

30

32

34

Time (min)

0

10

20

30

40

50

60

70

80

90

100

Relative Abundance

19.93

19.30

31.85

25.29

12.48

21.52

24.56

17.08

31.18

33.21

27.99

14.01

26.98

29.31

15.97

NL:

1.07E6

m/z=

395.18-

396.18

MS

0235lsmsf_

bison1

**Bison Sample 2 (0%)**

Time (min)

0237lsmsf_

D:\Update\...\0237lsmsf_bison2

11/13/2009 12:47:29 PM

Bison 2

RT:

12.00 - 35.00

SM:

7B

12

14

16

18

20

22

24

26

28

30

32

34

0

10

20

30

40

50

60

70

80

90

100

Relative Abundance

19.93

19.32

23.80

25.02

26.64

30.96

30.29

31.68

28.85

33.01

21.19

14.85

16.95

12.92

23.14

NL:

1.28E6

m/z=

395.18-

396.18

MS

bison2

NOTES

Assignment of peaks, with retention time(s) (RT) (min)

RT: 19.93 Hexacosanoate

**C_27_ *m/z* 407**

**Bison Sample 1 (39%)**

Time (min)

D:\Update\...\0235lsmsf_bison1

11/13/2009 11:06:22 AM

Bison 1

RT:

12.00 - 35.00

SM:

7B

12

14

16

18

20

22

24

26

28

30

32

34

0

10

20

30

40

50

60

70

80

90

100

Relative Abundance

17.75

31.50

32.17

29.40

33.79

19.31

25.92

28.26

21.80

23.55

12.44

15.05

15.96

NL:

7.76E4

m/z=

407.19-

408.19

MS

0235lsmsf_

bison1

**Bison Sample 2 (19%)**

Time (min)

D:\Update\...\0237lsmsf_bison2

11/13/2009 12:47:29 PM

Bison 2

RT:

12.00 - 35.00

SM:

7B

12

14

16

18

20

22

24

26

28

30

32

34

0

10

20

30

40

50

60

70

80

90

100

Relative Abundance

17.75

23.09

32.86

21.82

28.16

30.61

26.28

32.08

33.14

24.66

13.50

30.32

20.30

17.10

13.77

18.78

16.73

NL:

3.64E4

m/z=

407.19-

408.19

MS

0237lsmsf_

bison2

NOTES

Assignment of peaks, with retention time(s) (RT) (min)

RT: 17.75 Mycolipenate

**C_27_ *m/z* 409**

**Bison Sample 1 (9%)**

Time (min)

D:\Update\...\0235lsmsf_bison1

11/13/2009 11:06:22 AM

Bison 1

RT:

12.00 - 35.00

SM:

7B

12

14

16

18

20

22

24

26

28

30

32

34

0

10

20

30

40

50

60

70

80

90

100

Relative Abundance

21.81

17.20

21.32

19.93

28.05

33.23

12.41

17.71

32.07

29.58

26.19

14.19

14.99

25.23

23.43

19.56

15.93

NL:

4.80E4

m/z=

409.21-

410.21

MS

0235lsmsf_

bison1

**Bison Sample 2 (6%)**

D:\Update\...\0237lsmsf_bison2

11/13/2009 12:47:29 PM

Bison 2

RT:

12.00 - 35.00

SM:

7B

12

14

16

18

20

22

24

26

28

30

32

34

Time (min)

0

10

20

30

40

50

60

70

80

90

100

Relative Abundance

21.80

19.93

17.20

32.46

14.38

28.05

12.40

31.29

34.19

29.81

15.45

25.30

22.93

26.21

19.73

17.28

NL:

6.76E4

m/z=

409.21-

410.21

MS

0237lsmsf_

bison2

NOTES

Assignment of peaks, with retention time(s) (RT) (min)

RT: 17.20 C_27_ mycocerosate

19.93 Unknown

21.80 (21.81) Heptacosanoate

**C_29_ *m/z* 437**

**Bison Sample 1 (56%)**

D:\Update\...\0235lsmsf_bison1

11/13/2009 11:06:22 AM

Bison 1

RT:

12.00 - 35.00

SM:

7B

12

14

16

18

20

22

24

26

28

30

32

34

Time (min)

0

10

20

30

40

50

60

70

80

90

100

Relative Abundance

12.62

20.17

15.65

20.31

12.83

26.83

32.34

27.93

29.50

26.27

31.31

16.99

34.56

21.98

14.32

18.53

24.09

NL:

1.34E5

m/z=

437.26-

438.26

MS

0235lsmsf_

bison1

**Bison Sample 2 (42%)**

0237lsmsf_

Time (min)

437.26-

D:\Update\...\0237lsmsf_bison2

11/13/2009 12:47:29 PM

Bison 2

RT:

12.00 - 35.00

SM:

7B

12

14

16

18

20

22

24

26

28

30

32

34

0

10

20

30

40

50

60

70

80

90

100

Relative Abundance

12.62

20.17

15.62

26.81

20.31

32.58

14.54

32.16

31.04

27.47

34.35

22.12

15.81

23.25

13.01

26.39

16.97

18.45

21.47

NL:

5.75E4

m/z=

438.26

MS

bison2

NOTES

Assignment of peaks, with retention time(s) (RT) (min)

RT: 12.62, 15.65 (15.62) Unknowns

20.17 & 20.31 C_29_ mycocerosate (native & racemised)

26.81 Nonacosanoate

**C_30_ *m/z* 451**

**Bison Sample 1 (41%)**

D:\Update\...\0235lsmsf_bison1

11/13/2009 11:06:22 AM

Bison 1

RT:

12.00 - 35.00

SM:

7B

12

14

16

18

20

22

24

26

28

30

32

34

Time (min)

0

10

20

30

40

50

60

70

80

90

100

Relative Abundance

20.28

30.11

20.43

12.62

33.21

24.36

14.44

15.38

24.87

28.72

32.59

27.76

17.32

34.08

21.49

22.25

19.94

NL:

5.59E4

m/z=

451.29-

452.29

MS

0235lsmsf_

bison1

**Bison Sample 2 (30%)**

D:\Update\...\0237lsmsf_bison2

11/13/2009 12:47:29 PM

Bison 2

RT:

12.00 - 35.00

SM:

7B

12

14

16

18

20

22

24

26

28

30

32

34

Time (min)

0

10

20

30

40

50

60

70

80

90

100

Relative Abundance

30.13

20.28

20.43

13.37

34.30

32.96

25.02

29.78

15.12

21.92

18.52

32.48

23.19

28.75

15.39

25.70

NL:

4.67E4

m/z=

451.29-

452.29

MS

0237lsmsf_

bison2

NOTES

Assignment of peaks, with retention time(s) (RT) (min)

RT: 20.28 & 20.43 C_30_ mycocerosate (native & racemised)

30.11 (30.13) Triacontanoate

**C_32_ *m/z* 479**

**Bison Sample 1 (100%)**

Time (min)

D:\Update\...\0235lsmsf_bison1

11/13/2009 11:06:22 AM

Bison 1

RT:

12.00 - 35.00

SM:

7B

12

14

16

18

20

22

24

26

28

30

32

34

0

10

20

30

40

50

60

70

80

90

100

Relative Abundance

24.58

24.79

32.58

18.38

14.50

15.62

30.85

13.35

29.38

21.55

16.45

34.44

20.07

22.07

26.40

27.31

NL:

9.41E4

m/z=

479.34-

480.34

MS

0235lsmsf_

bison1

**Bison Sample 2 (100%)**

D:\Update\...\0237lsmsf_bison2

11/13/2009 12:47:29 PM

Bison 2

RT:

12.00 - 35.00

SM:

7B

12

14

16

18

20

22

24

26

28

30

32

34

Time (min)

0

10

20

30

40

50

60

70

80

90

100

Relative Abundance

24.57

24.78

29.88

13.51

28.04

34.26

33.75

17.84

25.70

32.21

24.33

18.88

14.05

15.44

21.52

NL:

6.94E4

m/z=

479.34-

480.34

MS

0237lsmsf_

bison2

NOTES

Assignment of peaks, with retention time(s) (RT) (min)

RT: 24.58 (24.57) & 24.79 (24.78) C_32_ mycocerosate (native & racemised)

**C_33_ *m/z* 493 & C_34_ *m/z* 507**

**Bison Sample 1 (0%, 0%)**

Time (min)

D:\Update\...\0235lsmsf_bison1

11/13/2009 11:06:22 AM

Bison 1

RT:

12.00 - 35.00

SM:

7B

12

14

16

18

20

22

24

26

28

30

32

34

0

20

40

60

80

100

0

20

40

60

80

100

Relative Abundance

17.97

26.74

32.32

21.04

18.36

23.40

24.59

17.81

33.24

30.88

28.74

13.20

16.11

19.56

24.99

13.00

15.02

27.39

16.57

28.21

30.92

19.77

17.82

31.49

33.29

13.44

25.56

23.58

21.06

NL:

1.54E4

m/z=

493.37-

494.37

MS

0235lsmsf_

bison1

NL:

1.71E4

m/z=

507.39-

508.39

MS

0235lsmsf_

bison1

**Bison Sample 2 (0%, 0%)**

Time (min)

D:\Update\...\0237lsmsf_bison2

11/13/2009 12:47:29 PM

Bison 2

RT:

12.00 - 35.00

SM:

7B

12

14

16

18

20

22

24

26

28

30

32

34

0

20

40

60

80

100

0

20

40

60

80

100

Relative Abundance

18.43

20.96

18.60

18.01

24.47

28.65

25.26

22.40

12.35

27.20

16.35

14.70

32.44

31.98

29.80

34.68

24.66

26.74

17.26

12.46

30.45

13.28

31.16

18.64

29.50

15.06

32.44

19.35

27.76

33.18

25.60

24.04

21.77

22.68

21.05

NL:

1.56E4

m/z=

493.37-

494.37

MS

0237lsmsf_

bison2

NL:

1.73E4

m/z=

507.39-

508.39

MS

0237lsmsf_

bison2

NOTES

Assignment of peaks, with retention time(s) (RT) (min)

No significant peaks
