# Supplementary material for: Mycobacterium tuberculosis Complex Lipid Virulence Factors Preserved in the 17,000-Year-Old Skeleton of an Extinct Bison, Bison antiquus
Source: PLoS One. 2012 Jul 30;7(7):e41923. doi: 10.1371/journal.pone.0041923 (PMC3408397; doi:10.1371/journal.pone.0041923)
Supplement: Figure S2 — Solvent system for the purification of pentafluorobenzyl (PFB) mycocerosates on C18 reverse phase cartridges. (DOC) [file pone.0041923.s002.doc]

**Figure S2** Solvent system for the purification of pentafluorobenzyl (PFB) mycocerosates on C_18_ reverse phase cartridges.

Alltech 205250 (500mg) C_18_ reverse phase cartridges were used. Each fraction (6 ml) was evaporated to dryness and fractions 6 and 7 were combined and analysed by NICI-GCMS.

| Fraction | Water | Methanol | Toluene |
| --- | --- | --- | --- |
| 1 | 1.8 | 4.2 | 0.0 |
| 2 | 1.1 | 4.9 | 0.0 |
| 3 | 1.0 | 5.0 | 0.0 |
| 4 | 0.9 | 5.1 | 0.0 |
| 5 | 0.8 | 5.2 | 0.0 |
| 6 | 0.0 | 6.0 | 0.0 |
| 7 | 0.0 | 6.0 | 0.0 |
| 8 | 0.0 | 4.8 | 1.2 |
