# Supplementary material for: Mycobacterium tuberculosis Complex Lipid Virulence Factors Preserved in the 17,000-Year-Old Skeleton of an Extinct Bison, Bison antiquus
Source: PLoS One. 2012 Jul 30;7(7):e41923. doi: 10.1371/journal.pone.0041923 (PMC3408397; doi:10.1371/journal.pone.0041923)
Supplement: Figure S3 — Solvent system for the purification of pyrenebutyric acid (PBA) derivatives of members of the phthiocerol family on C18 reverse phase cartridges. (DOC) [file pone.0041923.s003.doc]

**Figure S3** Solvent system for the purification of pyrenebutyric acid (PBA) derivatives of members of the phthiocerol family on C_18_ reverse reverse phase cartridges.

Alltech 205250 (500mg) C_18_ reverse reverse phase cartridges were used. Each fraction (6 ml) was evaporated to dryness and fractions 4, 5 and 6 were combined and analysed by HPLC.

| Fraction | Water | Acetonitrile | Dichloromethane |
| --- | --- | --- | --- |
| 1 | 3.0 | 3.0 | 0.0 |
| 2 | 1.2 | 4.8 | 0.0 |
| 3 | 0.6 | 5.4 | 0.0 |
| 4 | 0.0 | 6.0 | 0.0 |
| 5 | 0.0 | 5.4 | 0.6 |
| 6 | 0.0 | 4.8 | 1.2 |
| 7 | 0.0 | 3.0 | 3.0 |
| 8 | 0.0 | 0.0 | 6.0 |
