# Supplementary material for: Mycobacterium tuberculosis Complex Lipid Virulence Factors Preserved in the 17,000-Year-Old Skeleton of an Extinct Bison, Bison antiquus
Source: PLoS One. 2012 Jul 30;7(7):e41923. doi: 10.1371/journal.pone.0041923 (PMC3408397; doi:10.1371/journal.pone.0041923)
Supplement: Figure S4 — HPLC conditions for analysis of pyrenebutyric acid (PBA) derivatives of members of the phthiocerol family. (DOC) [file pone.0041923.s004.doc]

**Figure S4** HPLC conditions for analysis of pyrenebutyric acid (PBA) derivatives of members of the phthiocerol family.

HPLC analyses used a VWR Hitachi Elite Pump L-2130, LaChrom Autosampler L-2200, Column Oven L-2300, operating with an IF2 User detector and Foxy Jr. Fraction Collector; the profiles were detected by Fluorescence detector L-2480 and the data processed by Scientific Software Inc. EZChrom Elite, version 3.1.6 software. Reverse phase (Alltech 81412 Alltima C18, 3μm 50 x 4.6mm) and normal phase columns (Alltech 81414 Alltima Silica, 3μm 50 x 4.6mm) were used. HPLC analysis conditions are shown below. Samples were dissolved in HPLC grade heptane (20µl -1ml) and 0.1 to 20µl was injected. Solvent blanks were run after any positive sample to ensure that there was no carry-over of material.

| Conditions | Column | Gradient elution program |
| --- | --- | --- |
| Flow rate: 1ml/min  Detector: Excitation 342nm, emission 376nm | Reverse phase | Acetonitrile/tetrahydrofuran: 85:15 to 60:40 in 30min |
|  | Normal phase | Heptane/ethyl acetate: 99:1 for 1 min and 99:1 to 91:9 over 30 mins |
